# Interaction-Based Model to Predict Tensile Strength of Compacted Mixtures from Individual Component Data

**DOI:** 10.1021/acs.molpharmaceut.5c00709

**Published:** 2025-07-14

**Authors:** Pradeep Valekar, Ira S. Buckner

**Affiliations:** Graduate School of Pharmaceutical Sciences, 6613 Duquesne University, Pittsburgh, Pennsylvania 15282, United States

**Keywords:** tablet, tensile strength, particle−particle contacts, interaction-based models, cohesive, adhesive, binomial probability, geometric mean, pairwise interactions, higher-order interactions

## Abstract

The tensile strength of powder mixtures is a function of the strength of particle–particle contacts broken as the compact fails. Previous attempts to model compact strength based on interparticulate contacts have assumed pairwise interactions (1-to-1 interactions) between adjacent particles. This assumption, which originates from gaseous systems, may not adequately describe the behavior of consolidated systems, in which multiple adjacent particles interact simultaneously. In this study, interparticle contacts were modeled using higher-order interactions to predict the tensile strength of the compacted powder mixtures. Pairwise interactions are either completely cohesive or completely adhesive. The present model defines interactions in terms of multiple particles, leading to a distribution of distinct adhesive interactions whose strength is reflected by the composition of the interacting particle cluster. We have found that fourth-order interactions seem to produce the most accurate predictions, which is consistent with observations of interparticle interactions in consolidated systems. This model was evaluated using compacted mixtures containing diverse materials and compared to previously reported mixture prediction models. This new model produced superior predictions in all cases, with deviations between predicted and measured strength of ≤0.22 MPa on average.

## Graphical abstract

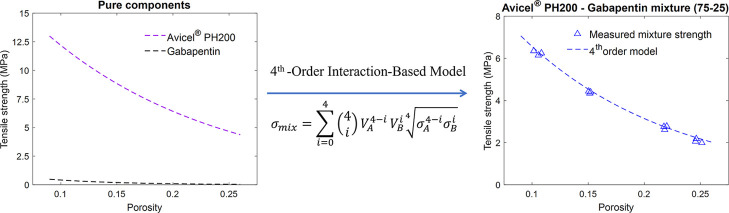

## Introduction

Tablet strength is a critical quality attribute that determines the success of the tableting process. An excessively strong tablet may fail disintegration and subsequent dissolution. On the other hand, a weaker tablet may result in an out-of-specification product in friability and tablet strength testing. Thus, optimizing the tablet strength is highly important to ensure the success of tableting unit operation. To aid in the early stages of tablet product development, it is important to assess the mechanical properties quickly. However, material availability tends to be limited at this stage. The bulk methods commonly used for mechanical property assessment require a relatively large amount of material. Late initiation of mechanical characterization studies usually delays formulation development. The prospective understanding of the mechanical properties of drug formulations by predictive methods can expedite decision-making at the early stages of development. This approach can help to guide the initial design space with a limited amount of material.^1^

Tablets are made by compaction of multicomponent mixtures comprised of drug and excipient powders. Each component contributes to the overall strength of the tablet. Mechanical property data of single components are generally available, and thus, it is feasible to predict the strength of mixtures from individual component data. Accurate predictions can enable the evaluation of different formulation compositions without the making of a large number of physical mixtures and tablets. As a result, different models have been proposed in the literature to make these predictions, including the linear,^2,3^ geometric,^2–5^ and Ryshkewitch-Duckworth-based mixing rules.^6–10^ These models allow the prediction of tensile strength of compacted mixtures from pure component profiles in select cases, although even the most accurate models proposed thus far have significant prediction errors when applied to more diverse sets of tableting materials.^8,11^

In addition to the above models, numerical solution methods such as the discrete element method (DEM) and finite element method (FEM) have been used to provide mechanistic insights into tableting processes. DEM has been used to provide particle-level insights into die fill,^12,13^ rearrangement,^14^ and densification^15^ during compaction. In contrast, FEM has been used to model the material deformation,^16^ density distribution,^17^ and failure mechanism of the tablets.^18,19^ To the best of the authors’ knowledge, these models have not been used to predict the tensile strength of formulation tablets directly from pure component properties. This study addresses this gap by introducing an interaction-based model for mixture strength prediction, which may be further enhanced by integrating it with numerical methods in future work.

Central to any prediction model of tablet strength is a mechanistic understanding of how particle–particle contacts contribute to the mechanical integrity of the tablet. The strength of a consolidated powder is a reflection of the nature of contacts broken during mechanical failure.^20^ Although some models are purely empirical, some efforts have been made to mechanistically incorporate the role of interparticle interactions. Current efforts^3,21–24^ to model compact strength based on interparticle contacts have been primarily based on the assumption of pairwise interactions, which has also been used to describe the behavior of gaseous systems.^25,26^ A pairwise interaction is defined completely by a pair of interacting particles. The overall behavior of the system is then approximated as a weighted average of all pairwise interactions.^27^ This assumption may not be justified in consolidated particulate systems, where each particle interacts with multiple adjacent particles simultaneously. These higher-order interactions between collections of particles lead to a more nuanced averaging of the component properties compared with pairwise averaging, where all interactions are categorized as purely adhesive (A–A) or purely cohesive (A–B). Higher-order interactions allow a spectrum of adhesive interaction types defined by the composition of a cluster of interacting particles. Therefore, the strength behavior of tablets might be more accurately predicted using a modeling strategy that considers higher-order interparticulate interactions.

In this study, we developed interaction-based models by extending the interactions to clusters containing a higher number of particles. In these models, it is assumed that an interaction cluster is defined by a specific number of particles where the order of the model corresponds to the number of interacting particles in a cluster. A separate version of the model can be produced for each order of interparticle interactions. In binary mixtures, the relative abundance of each cluster type is assumed to be described by the binomial probability distribution and the volume fraction of each material in the mixture. The strength of each cluster is assumed to be determined by the geometric mean of the component particle interaction strengths, based on the behavior in single component compacts. Various multicomponent mixtures were tested to assess their prediction performance and to identify the most accurate model.

## Theoretical Basis

The diametral compression test^28^ is generally used to assess the tensile strength of the tablets. In this test, the tablet is compressed diametrically until it fails in tension. Accordingly, the diametral compression test records the tensile stress required to initiate the fracture in the tablet. According to fracture mechanics, the failure involves the initiation of fracture at a flaw and propagation of fracture along the failure plane.^29^ At the microscopic level, tablets are aggregates of primary particles held together by particle–particle contacts. From here on, the particle–particle contacts will be referred to simply as contacts for brevity. The contacts present in the failure plane are broken during tensile failure. The crack-initiating flaw can be thought of as a collection of particles whose strength must be overcome to initiate the fracture. Thus, the tensile strength can be viewed as a function of the relative numbers and strengths of contacts broken within the strength-determining particle aggregate in which the flaw is formed during the test. Accordingly, theoretical approaches to modeling the tablet strength have been based on relating interaction strength at the particle level to the strength of the tablet.^21,22,30,31^ The elements of these previous attempts to model the tensile strength of mixtures in this way will be briefly described below as a foundation for the proposal of a new interaction-based tensile strength prediction model.

In pure component tablets, all contacts are cohesive. Assuming all particles are chemically equivalent, a single type of interaction strength (Φ_A_ or Φ_B_) determines the strength of the tablet (σ_A_ or σ_B_). In contrast, mixture tablets contain fractions of both cohesive and adhesive contacts. Different approaches have been used to estimate the strength of adhesive contacts (Φ_AB_). Amidon^3^ suggested that the arithmetic mean of the cohesive contact strengths might serve as a first approximation of the adhesive contact strength

1 $$\Phi_{AB}=\frac{1}{2}\left(\Phi_{A}+\Phi_{B}\right)$$

Even when proposed, the model (linear mixing rule) based on this equation was found to be inaccurate for some mixtures.^3^ The primary alternative for predicting adhesive strength from cohesive strength is the Berthelot rule.^32^ This rule states that adhesive interaction strength between two molecules can be approximated as the geometric mean of the cohesive interaction strengths of each type

2 $$\Phi_{AB}=\sqrt{\Phi_{A}\Phi_{B}}$$

Along with the strength of interparticle contacts holding the tablet together, the tensile strength is also determined by the number of contacts that are broken when failure of the tablets is initiated. In this context, the number of contacts is best defined in terms of the total area of contact between the particle surfaces. It is generally a reliable assumption to roughly relate the total contact area between particles to the solid fraction of the compacts. Not only does the solid fraction represent the fraction of the tablet’s volume that is occupied by solid particles, but it also represents the fraction of an average cross-section’s area that is filled with solid particles. Therefore, as the solid fraction increases, the amount of interparticle contact area that must be separated during breakage increases proportionally. Since the size of the element in which failure initiates is generally not known, the total amount of area being separated is not known either. Nevertheless, controlling the tablet’s solid fraction is a reasonable way to control the amount of interparticle contact area that determines the tablet’s tensile strength.

The formation of interparticle contacts is typically assumed to be a completely random process based on the relative amounts of surface area presented by the different types of particles. Amidon^3^ and Busignies et al.^23,24^ applied the binomial probability approach to calculate the relative fraction of each type of pairwise contact in binary mixtures (A–A, B–B, and A–B). According to this approach, each contact is a random sample of two particles from the population of mixed particles with the probability of sampling a given particle being determined by the volume fraction of that particle type in the mixture. Rather than surface area, the composition of the population was represented by the volume fraction of each component (*V*_A_ or *V*_B_), assuming the particles were similar in size and shape. According to this approach, the contributions of cohesive contacts to the overall strength become *V*_A_^2^ and *V*_B_^2^, for each material, and the contribution of adhesive contacts is 2*V_A_*V_B_.

Contacts can be broadly classified into cohesive and adhesive types. Most commonly, interactions are modeled either explicitly or implicitly by assuming that an interaction involves a pair of particles. In more general terms, this is known as second-order interaction. Chan,^22^ Amidon,^3^ and Busignies et al.^23,24^ modeled the type of contacts broken during tensile strength measurements using pairwise interactions (1-to-1 interactions) between adjacent particles. Figure 1 shows the schematics of pairwise contacts broken during tensile strength testing. In a binary mixture composed of components A and B, three pairwise contact types are possible: A–A, B–B, and A–B. The pairwise assumption considers only one adhesive contact type (A–B). A given particle can participate in more than one type of interaction simultaneously, in some cases,^21^ but each interaction would be considered a full contact with equal contribution to the mixture’s strength. For example, a particle forming an interaction with 3 neighboring particles across the fracture plane, as shown in the fourth-order model in Figure 1, would be assumed to have a larger influence on the strength of the tablet than a particle that only forms interactions with 2 even if all the particles have the same surface area available to form interactions.

**Figure 1:**
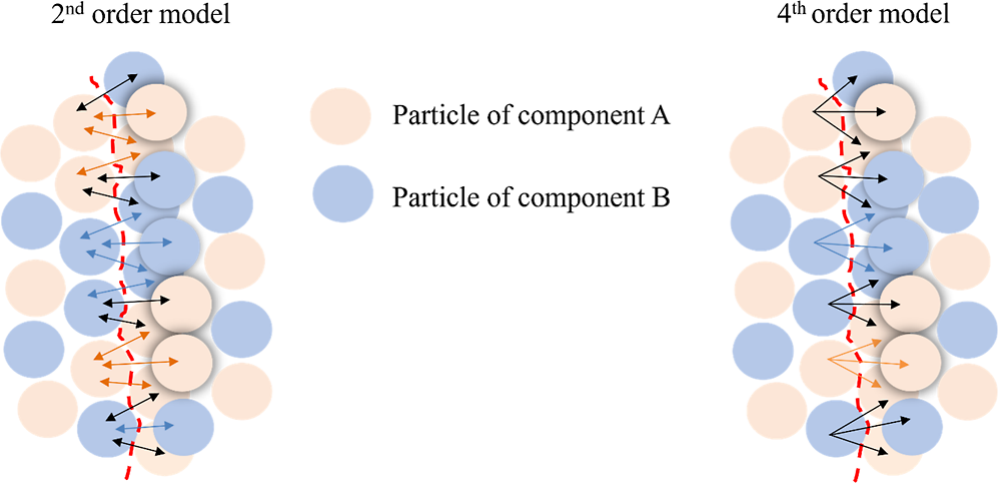
Schematics illustrating the breakage of interparticle contacts during tensile strength testing for the second-order model (double-sided arrows represent pairwise interactions) and the fourth-order model.

An alternative to describing all interactions as pairwise interactions was published by Busignies et al.,^23,24^ who applied third-order interactions (interaction clusters containing three particles). This application was aimed at fitting mixture data to determine empirical interaction parameters rather than making predictions of mixture behavior from component properties directly. Nevertheless, it is a prime example of the potential to use higher-order interactions in describing properties that are dependent on interparticle interactions.

The model being proposed is based upon (1) higher-order interactions better representing the relative contribution of each component to the overall strength and (2) the interaction strength of each particle cluster determined by the geometric mean of the interaction strengths of the individual particles involved. As with earlier models,^3,23,24^ the present model uses the binomial distribution to determine the probability of forming each type of particle cluster, assuming a random arrangement of particles in the powder bed. The interaction order directly determines the number of different types of adhesive interactions that are formed, which also determines the relative contribution of cohesive interactions compared with adhesive interactions. For example, a second-order model (pairwise interactions) predicts that 50% of the interactions will be cohesive in an equal-parts mixture of 2 components (*V*_A_^2^ + *V*_B_^2^), whereas a sixth-order model predicts that only 3% (*V*_A_^6^ + *V*_B_^6^) of the interactions will be purely cohesive. The impact of the order on the balance between cohesive and adhesive interactions in equal-part binary mixtures is illustrated by Figure 2, which compares the type and number fraction of each contact type predicted by second-, third-, fourth-, and sixth-order models. As the order of the model increases, more weight is given to adhesive contacts, and less weightage is given to the cohesive contacts regardless of how the particles are arranged. This reflects the behavior of binomial distribution. The central limit theorem leads to greater weight in the middle of the distribution as the size of the interaction cluster increases, so there is an expected relationship between the interaction order and the weight applied to adhesive interactions.

**Figure 2:**
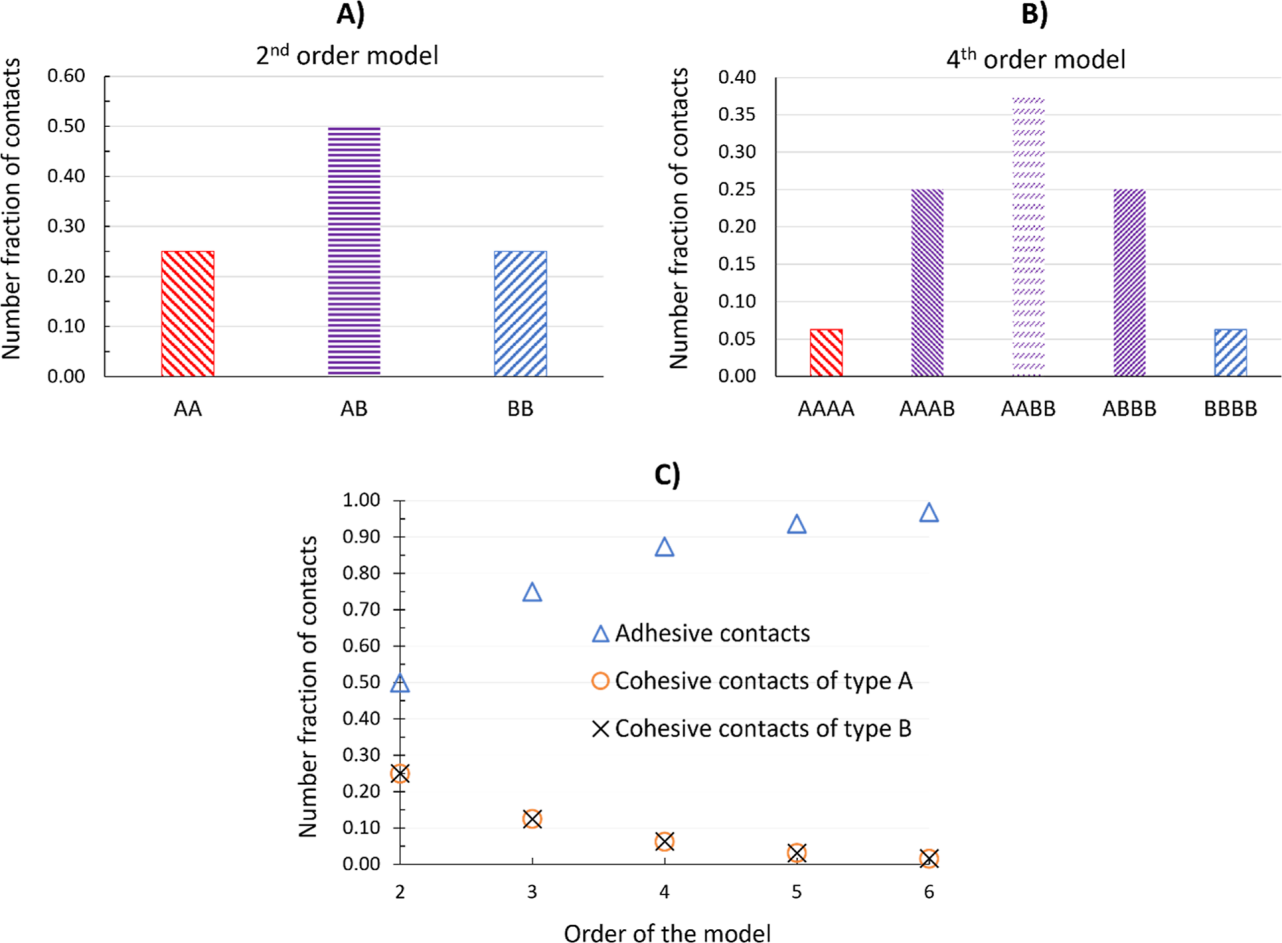
Different contact types for binary mixtures with 50–50 composition: (A) second-order model and (B) fourth-order model. (C) Number fraction of different contact types for second- to sixth-order models.

For binary mixtures, interaction-based models take the following general form

3 $$\sigma_{mix}=\sum_{i=0}^{n}\left(\begin{array}{l} n\\ i \end{array}\right)V_{A}^{n-i}V_{B}^{i}\sqrt[{n}]{\sigma_{A}^{n-i}\sigma_{B}^{i}}$$

where *n* represents the number of particles in the interaction cluster (order of interparticle interactions), *i* is the number of particles of B in the interaction cluster, *n* – *i* is the number of particles of A in the interaction cluster, *V* is the volume fraction of the components, and lastly, σ is the tensile strength of each component at the same solid fraction. It is important to recognize that the strength predicted by eq 3 represents the strength required to initiate fracture of the compact. It does not represent the sum of the strengths of all contacts ultimately broken in the failure plane.

By varying *n* in eq 3, a separate model can be obtained for each interaction order, ranging from *n* = 2 for pairwise interaction to ∞, which assumes that every particle interacts with every other particle. The present work will focus on the fourth-order model, which takes the form

4 $$\sigma_{mix}=V_{A}^{4}\sigma_{A}+V_{B}^{4}\sigma_{B}+4V_{A}^{3}V_{B}\sqrt[{4}]{\sigma_{A}^{3}\sigma_{B}}+6{V_{A}^{2}V}_{B}^{2}\sqrt[{4}]{\sigma_{A}^{2}\sigma_{B}^{2}}+4{V_{A}V}_{B}^{3}\sqrt[{4}]{\sigma_{A}\sigma_{B}^{3}}$$

We have found this particular model to consistently be the most accurate model for predicting the tensile strength in a diverse set of mixtures, and it is consistent with observations of the number of interparticle contacts broken per particle during failure. One study used particle coordination data from 113 reports and concluded that in the solid fraction range typically encountered in pharmaceutical tablets, the coordination of the average particle varied between 8 and 10.^33^ This would correspond to an interaction order ranging from 5 to 6, assuming that tensile failure of the tablet involves particles losing roughly half of their interactions due to separation along the failure plane. For the pharmaceutically relevant porosity range of 0.10 to 0.30, other studies have also concluded that 4 to 5 contacts are broken per particle during tensile strength measurement.^34–36^ Furthermore, little difference has been observed between the predictions generated by the fourth- and fifth-order models.

The utility of the fourth-order interaction-based model to predict mixture tensile strength will be demonstrated by using a variety of common pharmaceutical tableting excipients and drug substances. The accuracy of the model will be compared to previously reported models based on the root-mean-square error. Finally, the model will be used to generate a formulation design space for a simple formulation using only the compaction properties of the ingredients.

## Materials and Methods

### Pure Components

Five tableting excipients: Microcrystalline cellulose (Avicel PH 200, FMC Corporation, Philadelphia, PA), spray-dried lactose (316 grade, Foremost Farms, Baraboo, WI), a partially pregelatinized corn starch (Starch 1500, Colorcon, West Point, PA), dibasic calcium phosphate dihydrate (Emcompress, JRS Pharma, Germany), and EMDEX (JRS Pharma, Germany) and two drugs: aspirin (Spectrum Chemicals, Gardena, CA) and gabapentin (Hangzhou Starshine Pharmaceutical Co., Ltd., China) were used in this work. Both drugs, as received, had thick, elongated particles. Aspirin was milled using a coffee grinder (Encore ZCG485BLK, Baratza, WA). Gabapentin was milled using a conical screen mill (model 197S, Quadro Engineering, Canada). For excipients and milled drugs, a 90–250 μm sieve fraction was collected.

Pure components were characterized for particle size and shape with the objective of selecting materials with similar particle size and shape distributions. Micrographs were collected using an Olympus BX-51 optical microscope under 40× magnification. Additionally, dynamic image analysis was performed using Canty SolidSizer (J.M Canty Inc., Buffalo, NY). At least 2000 particles were analyzed to obtain the particle size and aspect ratio distributions.

### Binary Mixtures

It was observed in preliminary studies that when mixtures contained both components with similar tensile strengths, the predictions by all models converged. For such mixtures, any model can predict the tensile strength. The details of preliminary studies are reported in Supporting Information 1. Therefore, binary mixtures containing a stronger bonded material and a weaker material were studied. These mixtures are detailed in Table 1. Component 1 is the more strongly bonded material in each mixture. The ratio of the tensile strength of components at different porosities is also reported. For mixtures where at least one pure component was unable to form the tablet at a porosity of 0.10 or 0.15, the σ_1_/σ_2_ is not reported.

**Table 1:** Details of Binary Mixtures

			σ_1_/σ_2_			
#	component 1^a^	component 2^b^	ε = 0.10	ε = 0.15	ε = 0.20	ε = 0.25
1	Avicel PH 200	lactose		5.8	9.4	15.4
2	Avicel PH 200	Emcompress		5.0	8.0	12.6
3	Avicel PH 200	starch			7.5	10.3
4	Avicel PH 200	aspirin		9.0	8.8	8.6
5	Avicel PH 200	gabapentin		46.0	71.5	111.3
6	EMDEX	aspirin	7.1	3.7	2.6	1.8
7	EMDEX	gabapentin	36.3	18.8	20.9	23.2

a Stronger bonded material.

b Weaker bonded material.

Each mixture was prepared at 75–25, 50–50, and 25–75 volume fraction ratios. The mixtures were prepared at a batch size of 12 g using a two-step process to ensure homogeneity in the mixture. First, high-shear mixing was performed using a lab-scale mixer (model Lightnin TS2010, Mixing Equipment Co. Inc., USA) in a ∼60 mL glass container. The impeller was operated at 250 rpm for 5 min. This was followed by tumble mixing in the same container using a laboratory rotator (Model #10101, Appropriate Technical Resources (ATR), MD) at 15 rpm for 30 min. The ability of the two-step mixing process to produce homogeneous mixtures at an individual tablet level was confirmed using NIR chemical imaging (data not shown here).

### Volume Fraction of Components

Volume fraction was determined by using the weight fraction (*w*) and true density (ρ) of pure components^6–9^

5 $$V_{i}=\frac{\frac{w_{i}}{\rho_{i}}}{\sum_{i}\frac{w_{i}}{\rho_{i}}}$$

### Powder True Density

The true density of pure components was determined using a helium pycnometer (Model: SPY-6DC, Quantachrome Instruments, Boynton Beach, FL). Samples weighing approximately 6 to 8 g were analyzed in triplicates. The samples were purged for 30 min before data was collected. The true densities of materials were consistent with previous reports.^37,38^ For binary mixtures, true densities were calculated by using the true density and weight proportion of each pure component.

### Compaction

All of the powder materials (pure materials and mixtures) were first equilibrated at an ambient temperature (22–25 °C) over a saturated MgCl_2_.H_2_O solution (∼33% RH) prior to compaction. Tablets were prepared on a Presster compaction simulator (Natoli, MO—formerly MCC, NJ) emulating a rotary tablet press (HT-AP38-MSU, Elizabeth HATA international, PA) equipped with standard B tooling (13 mm round, flat-faced) at a press speed of 20 rpm. Precompression was not used. Tablets were prepared at the target weight of 500 mg. No internal lubricant was used in any of the tablet preparation. For the compaction of harder materials (Emcompress, Lactose 316, and gabapentin) and their binary mixtures, the tooling was lubricated by swabbing with a 5% (w/v) magnesium stearate suspension in methanol. The tablets were prepared in the porosity range of 0.10 to 0.30. Three replicate tablets were prepared at each porosity.

### Tablet Porosity

After ejection, the tablets were stored in a desiccator under controlled humidity (∼33% RH) and ambient temperature conditions for at least 48–72 h to allow complete viscoelastic relaxation. The dimensions of each tablet were measured using a digital caliper (Mitutoyo, Japan). The weight (*W*), diameter (*D*), thickness (*t*), and powder true density (ρ) of each tablet were used in the following equation to obtain the porosity (ε).

6 $$\varepsilon =1-\frac{4W}{\pi D^{2}t\rho }$$

### Tablet Tensile Strength

Following the dimensional relaxation of tablets, tensile strength measurements were obtained on an Instron universal testing system (Model 5869, Norwood, MA) fitted with a 1 kN load cell. The setup consisted of two thin rectangular metal gauges, the width of which was less than one-sixth of the tablet’s diameter. The metal gauges were covered with two strips of blotting paper to reduce compressive and shear stresses at the points of contact with the tablets. The tablets were diametrically compressed between gauges at a cross-head speed of 5 mm/min. The force (*F*) required to cause the tensile failure of the tablets was recorded. The diametral tensile strength (σ) was obtained using the relaxed diameter (*D*) and thickness (*t*) of the tablet in the following equation

7 $$\sigma =\frac{2F}{\pi Dt}$$

The tensile strength and porosity data of pure components were fit with the Ryshkewitch-Duckworth (R-D) equation^39,40^ to obtain the full continuous compactibility profiles.

8 $$\sigma =\sigma_{0}e^{-k\varepsilon }$$

where σ is the tensile strength at porosity ε, σ_0_ is the tensile strength at zero porosity, and *k* is a constant representing bonding capacity.

### Prediction of Tensile Strength of Mixtures from Pure Component Data

Compactibility profiles and volume fractions of pure components were used as input parameters in interaction-based models to predict compactibility of binary mixtures. The predicted tensile strength was compared with the measured tensile strength to assess the performance of these models. This comparison was performed for all mixtures. The prediction performance of interaction-based models was also compared against the linear mixing rule, R-D-based rules, power law (geometric mixing rule), and compressibility-based geometric mixing rule. It is important to note that the linear mixing rule is a second-order (pairwise) model,^3^ where adhesive contact strength is determined by eq 1. The power law is explained by Etzler et al.^4^ and Amidon.^3^ The compressibility-based geometric mixing rule is described by Reynolds et al.^5^ The details of R-D-based rules can be found elsewhere.^8–10^

The models were also tested against the mixture data set collected from 6 published studies.^6,7,9,41–43^ The studies that reported the compactibility data for both the pure components and their corresponding mixtures were selected. In some studies, pure component data were derived from reported R-D parameters.^6,7,9^ When the studies did not report these parameters directly, figures containing pure component data were uploaded into WebPlotDigitizer (Rohatgi^44^), a Web-based tool for extracting quantitative data from images. The plot axes were first calibrated, followed by manually marking the individual data points to retrieve their x- and y coordinates. The mixture data from all studies were extracted using the same procedure. The accuracy of the digitized values was verified by comparing the extracted points with visually identifiable coordinates on the original figures.

Root-mean-square error (RMSE) was used to assess the accuracy of each model. It is a measure of the average difference between the predicted and measured values. A lower RMSE indicates a closer agreement between the prediction by the specific model and the experimental data. It was obtained using the following equation:

9 $$RMSE=\sqrt{\frac{\sum_{i=1}^{n}{\left(\sigma_{Measured}-\sigma_{Predicted}\right)}^{2}}{n}}$$

where *n* is the number of experimental data points. The RMSE was calculated for each individual mixture profile and globally across the overall data set to evaluate the performance on both individual mixtures as well as the overall collection of mixtures.

## Results and Discussion

### Pure Component Powders

The assumption of the binomial distribution is that each particle has an equal probability of being in a cluster, which is true if all particles have the same size and shape. It is important to recognize that the particles of pharmaceutical materials are not uniform in size and shape.^45^ Therefore, the objective was to identify materials with similar particle shapes and sizes. Using the aspect ratio as the indicator of the particle shape, the materials having aspect ratios closer to 1 were selected. Microscopy was used to screen the materials. Figure 3 shows the micrographs of the screened pure components.

**Figure 3:**
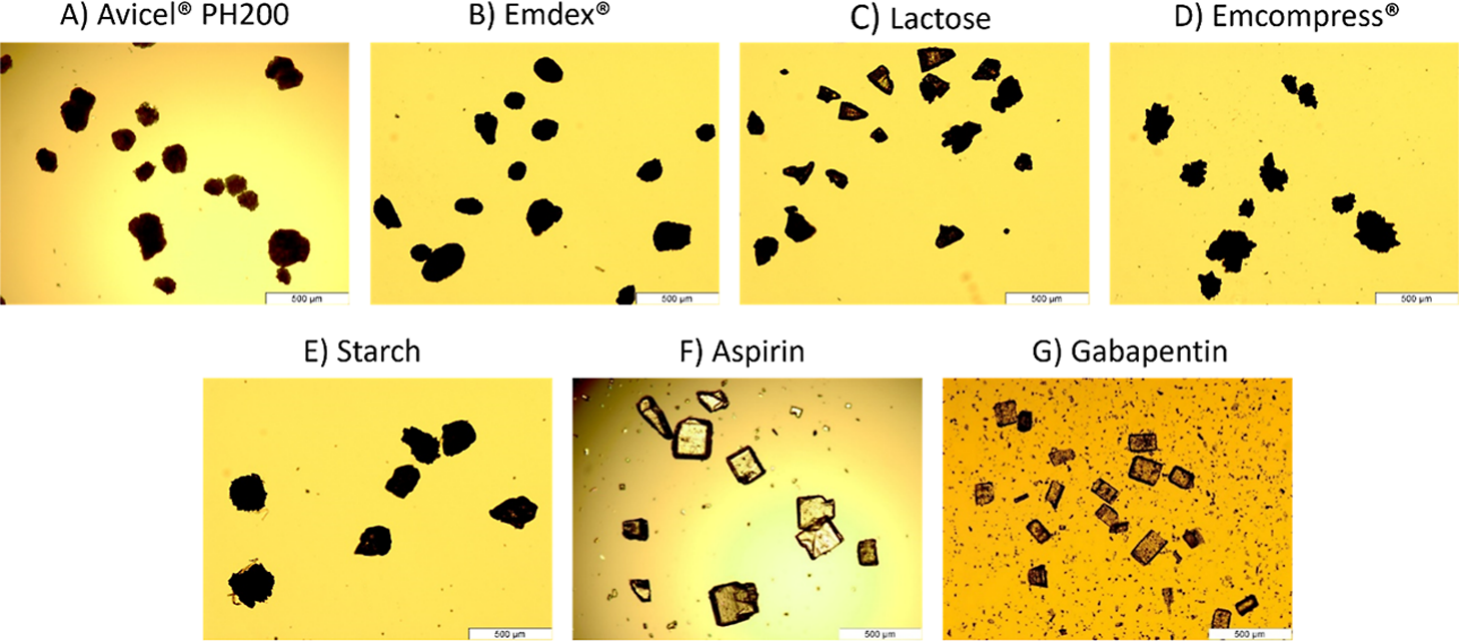
Micrographs of pure components.

As a technique, microscopy is limited by the number of particles that can be analyzed.^46^ To overcome this limitation, dynamic image analysis was performed. At least 2000 particles were analyzed for each pure component. Figure 4 shows the overlay of particle size and aspect ratio distributions of all components. Consistent with the assumption of the model, all materials showed similar particle size and aspect ratio distributions.

**Figure 4:**
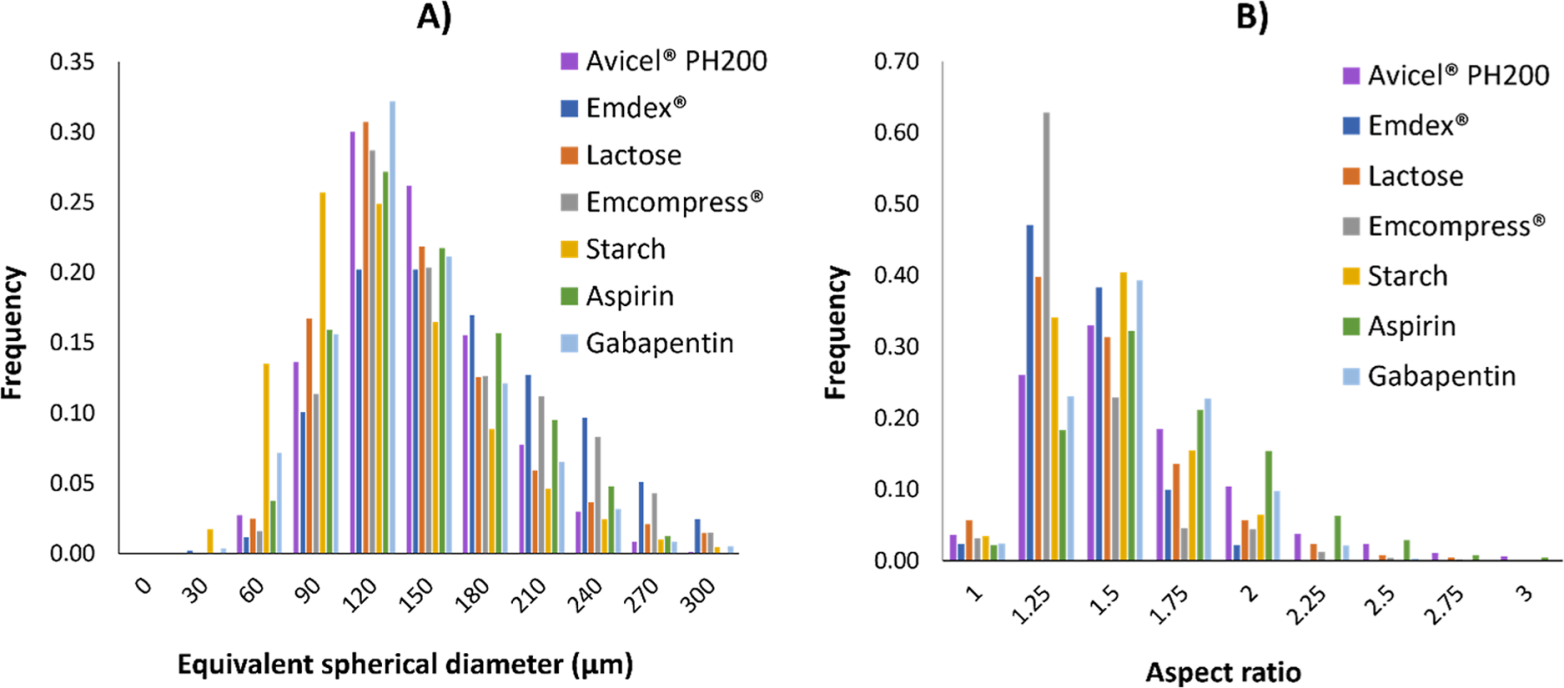
Overlay of (a) equivalent spherical diameter and (b) aspect ratio of pure components.

Figure 5 shows the variation in the tensile strength with porosity for the tablets made of individual powders considered in this study. These materials were selected to represent the wide range of compactibility behaviors. Avicel PH 200 and EMDEX were the materials with a higher compactibility. In contrast, aspirin, gabapentin, and starch showed lower compactibility. Lastly, lactose and Emcompress showed intermediate compactibility behaviors. In preliminary studies (Supporting Information 1), it was observed that binary mixtures containing components with higher strength differences are model systems for assessing the prediction performance of different versions of interaction-based models. Based on this observation, seven such binary combinations of pure components were used to evaluate the accuracy of interaction-based models.

**Figure 5:**
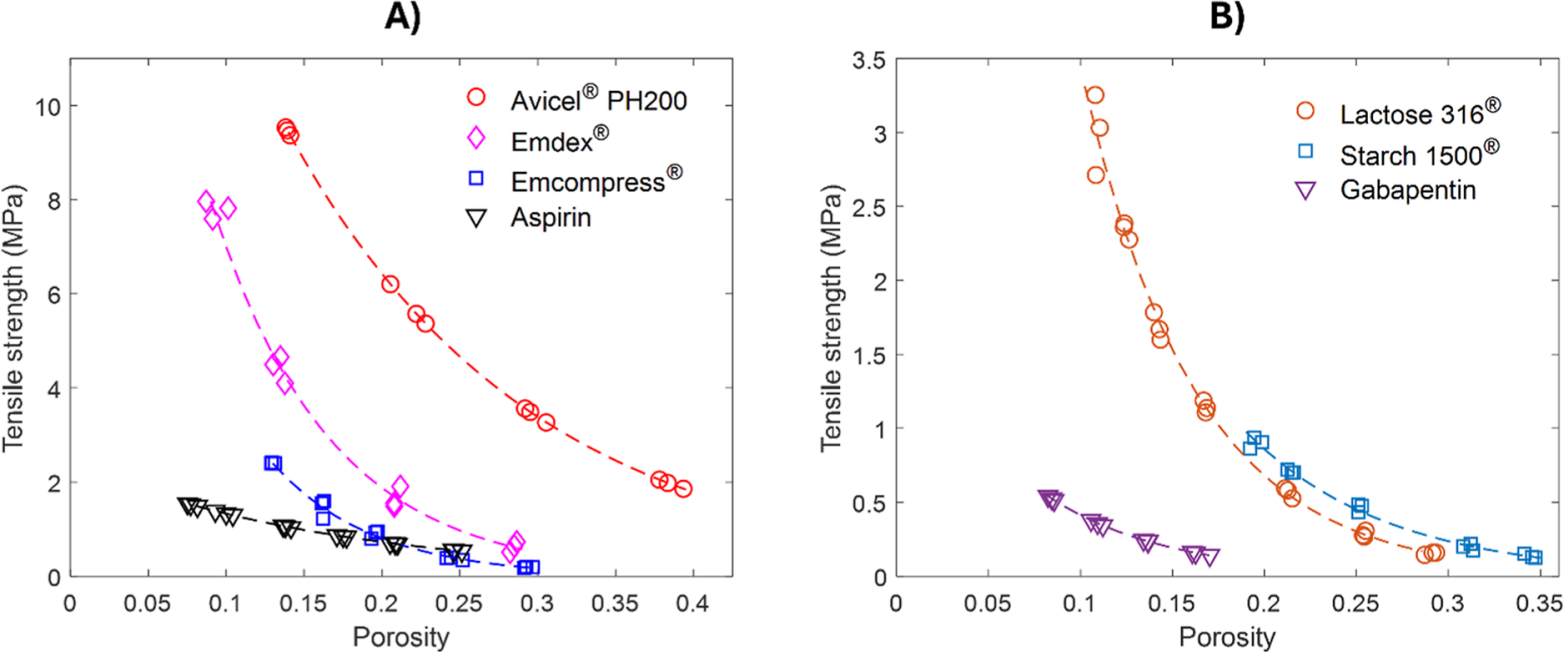
Compactibility profile of pure components. The dashed lines represent the fit to eq 8 for the corresponding material.

### Prediction of Tensile Strength of Binary Mixtures

The volume fraction and R-D parameters of the pure components were used as input parameters in different models to predict the compactibility profiles of the binary mixtures. The predicted compactibility profiles were compared to the experimental compactibility of the mixtures. For all binary mixtures analyzed using the new model in this study, fourth-order interactions showed the most accurate prediction of mixture strength. Figure 6 compares the strength predicted by the fourth-order model with the measured strength for Avicel PH 200-based binary mixtures at different compositions. The model predicted the tensile strength profile of these mixtures with RMSE ≤0.18 MPa. No systematic deviations were observed between predicted and measured values for each mixture at different compositions and different porosities.

**Figure 6:**
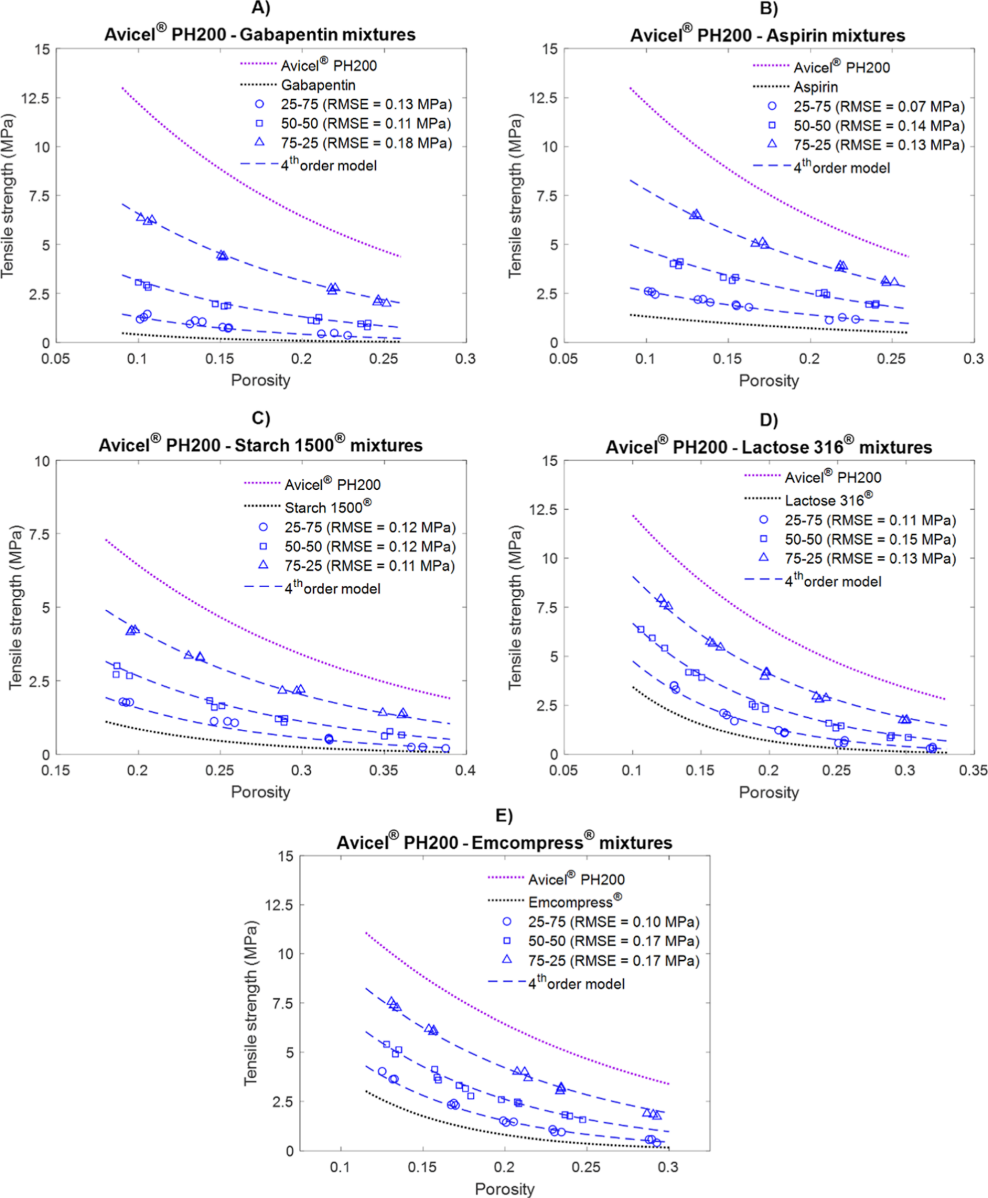
Comparison of measured and predicted strength by the fourth-order model for Avicel PH 200-based binary mixtures at different compositions. The dotted lines represent the pure components. The hollow markers represent the experimentally measured profile of the corresponding mixtures. The blue dashed lines represent the predictions by the fourth-order model.

To evaluate if the choice of the stronger bonded component in the mixture has any influence on the accuracy of the predictions, additional binary mixtures containing another stronger material, EMDEX were also tested. Figure 7 shows the comparison of predicted and measured strength of EMDEX-based binary mixtures at three different compositions and porosity levels. The model predicted the compactibility of these mixtures with RMSE ≤0.16 MPa. This suggests that the prediction performance of the model is not significantly affected by the choice of the stronger bonded material.

**Figure 7:**
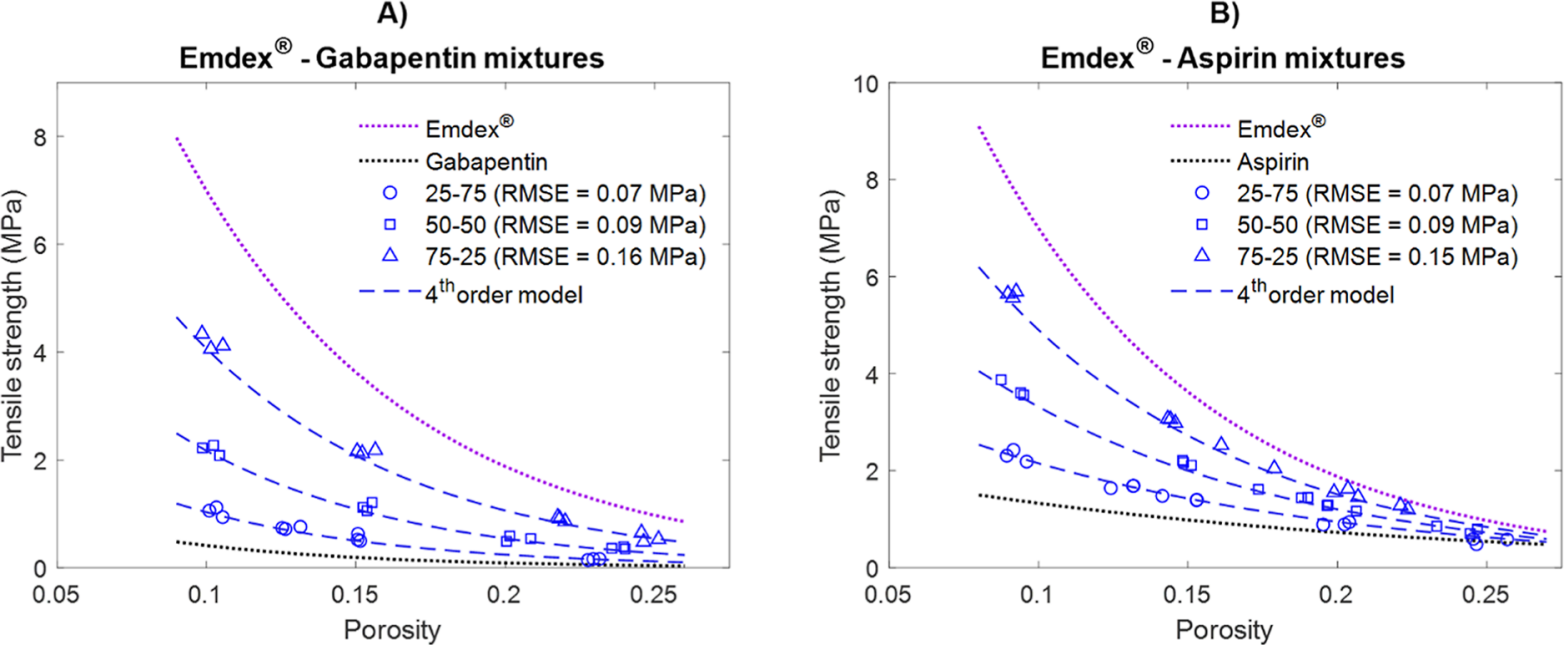
Comparison of measured and predicted strength by the fourth-order model for EMDEX-based binary mixtures at different compositions. The dotted lines represent the pure components. The hollow markers represent the experimentally measured profiles of the corresponding mixtures. The blue dashed lines represent the predictions by the fourth-order model.

Figure 8 shows the overall RMSE values of the different models applied to the compiled data set comprising 7 binary mixtures across all studied compositions and porosities. Again, the fourth-order model showed the best prediction performance compared to other models, as indicated by the lowest overall RMSE of 0.13 MPa. Overall, for individual mixtures as well as the compiled data set, the fourth-order model predicted the tensile strength with RMSE ≤0.18 MPa. The fifth-order model showed a similar performance with marginally higher RMSE (Figure 8). Regarding the physical meaning of the model order, fourth- and fifth-order interactions imply that on average, 3 and 4 contacts, respectively, are broken per particle during the tensile strength measurement. This is in close agreement with the metallurgical studies^34–36^ where, on average, 4 to 5 contacts were found to be broken per particle during strength testing for tablets consolidated in the pharmaceutically relevant porosity range. Although these studies are not based on pharmaceutical materials, they suggest that the present findings are reasonable.

**Figure 8:**
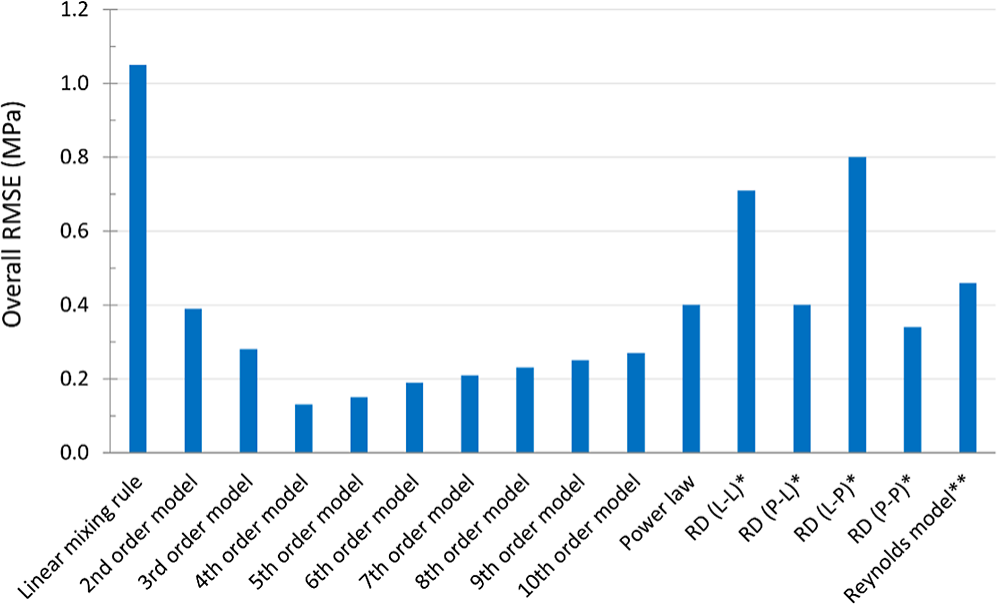
Overall RMSE for different models. * Models based on the application of power law (P) and linear mixing rule (L) to the Ryshkewitch-Duckworth (RD) equation. The first letter in the bracket refers to the law used to predict σ_0mix_ and the second letter refers to the law used to predict *k*_mix_. The details of these models can be found elsewhere.^6–10^ ** Model proposed by Reynolds et al.^5^

The comparison of the tensile strengths predicted by the new models to earlier models revealed an interesting trend. Figure 9 shows the overlay of tensile strength predictions by different models for the equal volume Avicel PH 200–gabapentin mixture. The linear mixing rule predicted the strongest strength among all models. In contrast, the power law predicted the lowest strength. The magnitudes of tensile strength predicted by different models were in the following order: Linear mixing rule > second-order > third-order > fourth-order > fifth-order...10th-order > power law.

**Figure 9:**
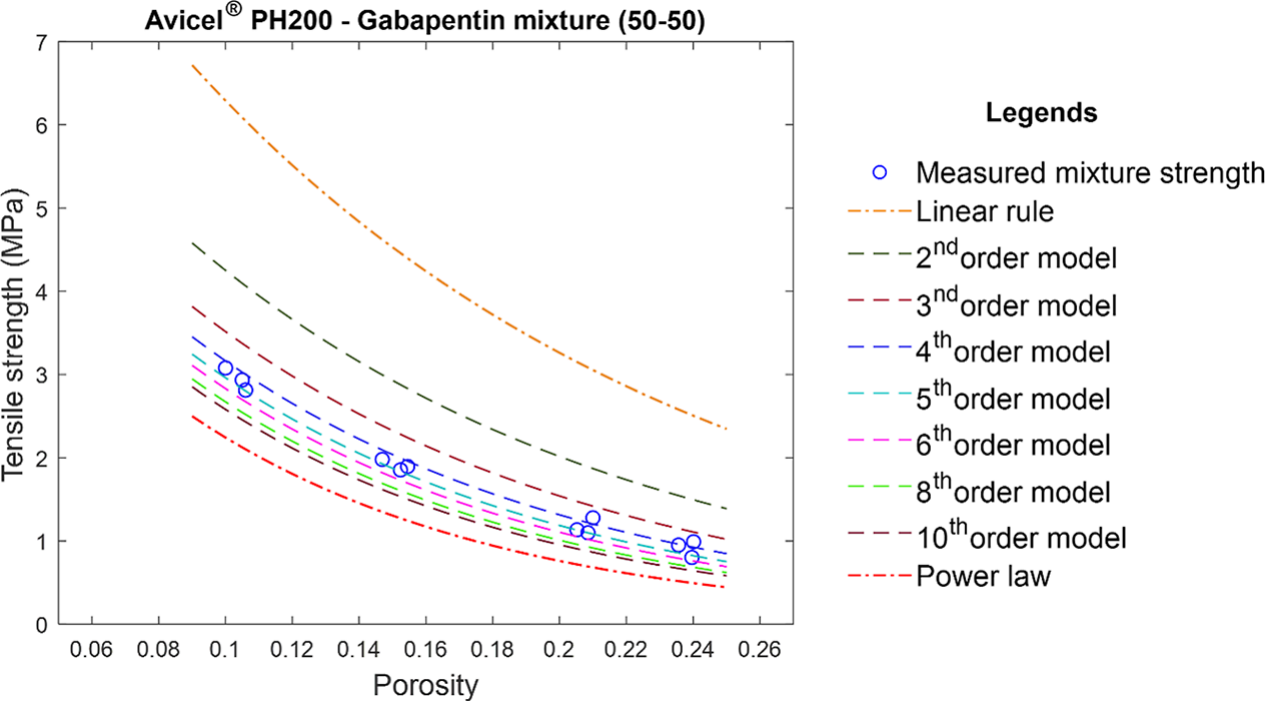
Overlay of tensile strength predictions by different models for the Avicel PH 200–gabapentin (50–50) mixture. The blue circle represents the experimentally measured profile of the corresponding binary mixture. The dashed lines represent the predictions by different models.

All binary mixtures used in this study followed the same rank order in their predictions. The reasons for the observed rank order of the predicted strength can be understood by focusing on a single porosity. Figure 10 shows a plot of the strengths predicted for the equal volume Avicel PH 200–gabapentin mixture at a porosity of 0.15, which is representative of all other mixtures and porosities. It is not surprising that the linear mixing rule always predicts a higher strength than the power law due to the mathematical relationship between the arithmetic and geometric means. It is more interesting that the interaction-based model predictions fall between the linear rule and the power law, gradually decreasing as the interaction order increases. Furthermore, the interaction-based model appears to asymptotically approach the power law as the interaction order becomes infinite. This trend can be understood by the relative weight that each model gives to purely cohesive interactions, in comparison to adhesive interactions. As previously described, the linear mixing rule applies the most weight to cohesive interactions, and the weight of cohesive interactions decreases as the interaction order or the number of particles involved in each interaction cluster increases. Etzler et al.^4^ justified the use of the power law by referencing the application of Berthelot’s rule^32^ to predicting interfacial tensions from the geometric mean of the surface tensions (equations 19 and 20 in Etzler et al.^4^). The interfacial tension exclusively reflects adhesive interactions between dissimilar molecules across the interface. In other words, the power law models the tensile strength as if all interactions were adhesive in nature. Therefore, it is reasonable that the interaction-based model predictions would approach the power law as the interaction order increases, and the relative weight that the cohesive interactions receive becomes vanishingly small. As can be seen in Figure 10, the measured strength of the mixture agrees most closely with an interaction order of 4 or 5 for this mixture. Overall, the current set of mixtures was most accurately predicted using fourth-order interactions (see Figures 6–8). This result suggests this interaction-cluster size most accurately reflects the relative balance between cohesive and adhesive interactions in determining the strength of compacted mixtures.

**Figure 10:**
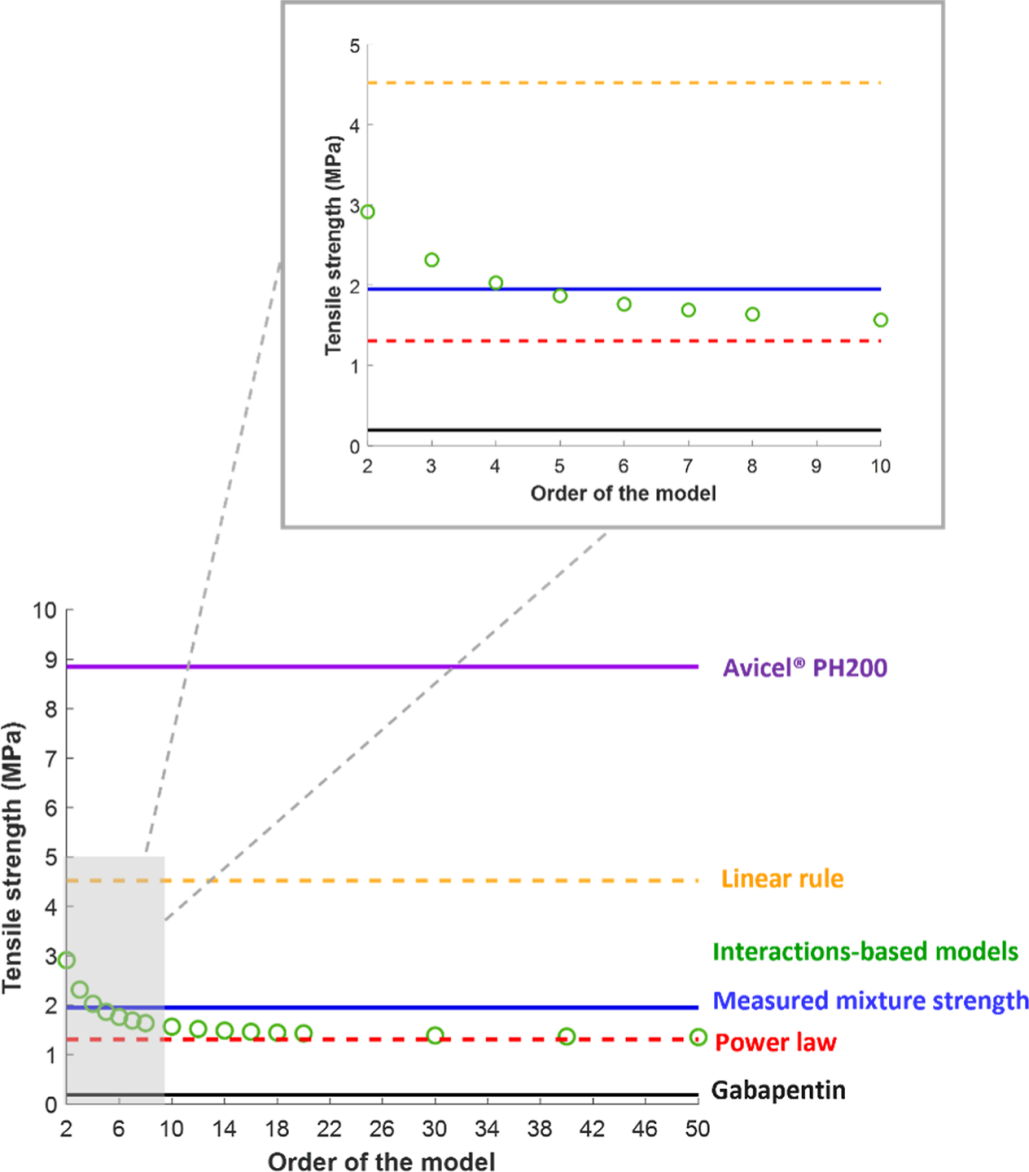
Variation in tensile strength as a function of the order of the model for the Avicel PH 200–gabapentin mixture (50–50) at the porosity of 0.15. The measured mixture strength, pure component strengths, the strengths predicted by the linear mixing rule, and power law at the same porosity are also shown for reference.

The main objective of this study was to develop a model that can accurately predict the tensile strength of a wide variety of compacted mixtures. Compared to other interaction orders, the fourth-order model showed the lowest RMSE for individual mixtures as well as the compiled data set. Hence, this model was selected to test the broader applicability of this model to an independent data set from published studies.^6,7,9,41–43^ The data set contained binary, ternary, and quaternary mixtures. To extend the model to multicomponent mixtures, trinomial and quadrinomial probability approaches were applied to the ternary and quaternary mixtures, respectively. The resultant equations of the above models and calculations are shown in Supporting Information 2. Again, the RMSE was used to assess the accuracy of the predictions.

The RMSE values for the application of the fourth-order interaction model to individual mixtures from the literature studies are reported in Table 2. All binary mixtures except Avicel PH 102–HPMC were predicted with RMSE ≤0.29 MPa. The reasons for the higher deviation in this mixture are unclear at this point. The ternary and quaternary mixtures were predicted with RMSE ≤0.21 MPa. The performance of the model in predicting ternary and quaternary mixtures is encouraging given that the end application is the prediction of actual direct compression formulations. Future studies may explore how robust this model is to particle size and shape differences in comparison to other models, which would also be impacted by nonrandom particle mixing associated with these differences.

**Table 2:** Prediction Performance of the Fourth-Order Model in Predicting the Tensile Strength of Individual Mixtures from Literature Data

mixture	number of compositions, porosity range	RMSE (MPa)	study
Avicel PH 102–Starch 1500	3, 0.10–0.48	0.25	Wu et al.^6^
Avicel PH 102–HPMC^a^	3, 0.16–0.46	0.47	
Avicel PH 102–lactose monohydrate NF	1, 0.08–0.48	0.25	Tye et al.^42^
Avicel PH 112–ibuprofen	1, 0.05–0.24	0.13	Patel and Bansal^9^
Emcompress–ibuprofen	1, 0.05–0.19	0.26	
Avicel PH 102–carbamazepine	4, 0.13–0.44	0.18	Mishra dissertation^43^
Avicel PH 102–Lactose 316	4, 0.09–0.47	0.29	
Chitosan–xanthan gum	4, 0.15–0.45	0.20	Eftaiha et al.^41^
Avicel PH 102–HPMC–Starch 1500	2, 0.13–0.40	0.18	Wu et al.^7^
Avicel PH 102–HPMC–Pharmatose 50M	2, 0.12–0.47	0.18	
Avicel PH 102–HPMC–Starch 1500–Pharmatose 50M	2, 0.13–0.45	0.21	

a Hydroxypropyl methylcellulose.

## Conclusion

A novel approach was used to model the tensile strength of the compacted mixtures. This approach models the types of contacts broken during strength measurements, assuming higher-order interactions between adjacent particles. The relative number and strength of individual interaction types were determined by binomial probability and the geometric mean rule, respectively. A separate model version of the model was obtained for each order of interparticle interactions. Binary mixtures containing components with diverse mechanical behaviors were tested to identify the most accurate model. The porosity for binary tablets ranged from 0.09 to 0.39 and exhibited tensile strengths between 0.2 and 8 MPa. The fourth-order model provided the most accurate predictions with an RMSE of ≤0.18 MPa for binary mixtures containing components with distinct compactibilities. The applicability of this model was also tested against the binary mixture data set collected from literature studies. The model predicted most of these mixtures with an RMSE of ≤0.29 MPa. The model was also applied to ternary and quaternary mixtures. These mixtures were predicted with RMSE ≤0.21 MPa, indicating the suitability of the model to predict the compactibility profiles of multicomponent mixtures.

This study has demonstrated that an interaction-based model produces very accurate predictions of mixture strength and is fundamentally consistent with physical observations of the interparticle interaction cluster behavior during compact failure. At the early stages of development, the model can be used to streamline the formulation screening in a material-sparing manner. Through this screening, the influence of the excipient grade, drug loading, and different porosities can be explored to find the composition space that meets the tensile strength criteria.

## Supplementary Material





## Notes

Prediction of tensile strength of mixtures containing components with varied strength differences by interactions-based models (PDF)
Excel template configured for the fourth-order model calculations for binary, ternary, and quaternary mixtures in which users can input the RD parameters, weight fraction, and true density of pure components in relevant cells and the template then predicts the tensile strength of the mixture (XLSX)

## References

[1] Kalaria D. R., Parker K., Reynolds G. K., Laru J. (2020). An industrial approach
towards solid dosage development for first-in-human studies: Application
of predictive science and lean principles. Drug
Discovery Today.

[2] Amidon, G. E. ; Secreast, P. J. ; Mudie, D. Particle, powder, and compact characterization. In Developing solid oral dosage forms; Elsevier, Inc., 2009; pp 163–186.

[3] Amidon, G. E. Compaction and the properties of mixtures; Compaction Simulation Forum: Cambridge, MA, 2012 November 13–14.

[4] Etzler F. M., Bramante T., Deanne R., Sienkiewicz S., Chen F. (2011). Tablet tensile strength: an adhesion science perspective. J. Adhes. Sci. Technol..

[5] Reynolds G. K., Campbell J. I., Roberts R. J. (2017). A compressibility
based model for
predicting the tensile strength of directly compressed pharmaceutical
powder mixtures. Int. J. Pharm..

[6] Wu C.-Y., Best S. M., Bentham A. C., Hancock B. C., Bonfield W. (2005). A simple predictive
model for the tensile strength of binary tablets. Eur. J. Pharm. Sci..

[7] Wu C.-Y., Best S. M., Bentham A. C., Hancock B. C., Bonfield W. (2006). Predicting
the tensile strength of compacted multi-component mixtures of pharmaceutical
powders. Pharm. Res..

[8] Michrafy A., Michrafy M., Kadiri M. S., Dodds J. A. (2007). Predictions
of tensile
strength of binary tablets using linear and power law mixing rules. Int. J. Pharm..

[9] Patel S., Bansal A. K. (2011). Prediction of mechanical properties
of compacted binary
mixtures containing high-dose poorly compressible drug. Int. J. Pharm..

[10] Jolliffe H. G., Ojo E., Mendez C., Houson I., Elkes R., Reynolds G., Kong A., Meehan E., Becker F. A., Piccione P. M. (2022). Linked experimental
and modelling approaches for tablet property
predictions. Int. J. Pharm..

[11] Puckhaber D., Voges A.-L., Rane S., David S., Gururajan B., Finke J. H., Kwade A. (2023). Enhanced multi-component model to
consider the lubricant effect on compressibility and compactibility. Eur. J. Pharm. Biopharm..

[12] Wu C.-Y. (2008). DEM simulations
of die filling during pharmaceutical tabletting. Particuology.

[13] Mateo-Ortiz D., Muzzio F. J., Méndez R. (2014). Particle size
segregation promoted
by powder flow in confined space: The die filling process case. Powder Technol..

[14] Martin C., Bouvard D., Shima S. (2003). Study of particle
rearrangement during
powder compaction by the discrete element method. J. Mech. Phys. Solids.

[15] Giannis K., Schilde C., Finke J. H., Kwade A. (2021). Modeling of
high-density
compaction of pharmaceutical tablets using multi-contact discrete
element method. Pharmaceutics.

[16] Desbois L., Tchoreloff P., Mazel V. (2020). Characterization and
modeling of
the viscoelasticity of pharmaceutical tablets. Int. J. Pharm..

[17] Diarra H., Mazel V., Busignies V., Tchoreloff P. (2015). Investigating
the effect of tablet thickness and punch curvature on density distribution
using finite elements method. Int. J. Pharm..

[18] Podczeck F., Drake K. R., Newton J. M. (2013). Investigations
into the tensile failure
of doubly-convex cylindrical tablets under diametral loading using
finite element methodology. Int. J. Pharm..

[19] Shang C., Sinka I., Pan J. (2013). Modelling
of the break force of tablets
under diametrical compression. Int. J. Pharm..

[20] Hiestand, E. N. Mechanics and Physical Principles for Powders and Compacts; SSCI, 2002.

[21] Cheng D.-H. (1968). The tensile
strength of powders. Chem. Eng. Sci..

[22] Chan S., Pilpel N., Cheng D.-H. (1983). The tensile
strengths of single powders
and binary mixtures. Powder Technol..

[23] Busignies V., Leclerc B., Porion P., Evesque P., Couarraze G., Tchoreloff P. (2006). Investigation
and modelling approach of the mechanical
properties of compacts made with binary mixtures of pharmaceutical
excipients. Eur. J. Pharm. Biopharm..

[24] Busignies V., Evesque P., Porion P., Leclerc B., Tchoreloff P. (2009). Mechanical
properties of compacts made with binary mixtures of pharmaceutical
excipients of three different kinds. AIP Conf.
Proc..

[25] Boltzmann, L. Lectures on Gas Theory, Translated by Stephen G. Brush; University of California Press: Berkeley, CA, 1964.

[26] Brilliantov, N. V. ; Pöschel, T. Kinetic Theory of Granular Gases; Oxford University Press: USA, 2004.

[27] van
der Waals J. D. (1898). Sur le Mélange des Gaz. Compt. Rendus.

[28] Fell J., Newton J. (1970). Determination of tablet strength by the diametral-compression
test. J. Pharm. Sci..

[29] Griffith, A. A. Theory of rupture. In Proceedings of the first International Congress for Applied Mechanics, Delft, 1924; J. Waltman, jr.: Delft, 1925; pp 55–63.

[30] Eriksson M., Alderborn G. (1995). The effect of particle fragmentation
and deformation
on the interparticulate bond formation process during powder compaction. Pharm. Res..

[31] Hiestand E. (1991). Tablet bond.
I. A theoretical model. Int. J. Pharm..

[32] Berthelot D. (1898). Sur le Mélange
des Gaz. Compt. Rendus.

[33] German R. M. (2014). Coordination
number changes during powder densification. Powder Technol..

[34] Arzt E. (1982). The influence
of an increasing particle coordination on the densification of spherical
powders. Acta Metall..

[35] Fischmeister H. F., Arzt E., Olsson L. (1978). Particle deformation
and sliding
during compaction of spherical powders: a study by quantitative metallography. Powder Metall..

[36] Fischmeister H. F., Arzt E. (1983). Densification of powders by particle
deformation. Powder Metall..

[37] Roopwani, R. Role of mechanical stress, excipients and coprocessing on tablet mechanical properties. Doctoral dissertation, Duquesne University, Pittsburgh, PA, 2015.

[38] Katz, J. M. Multi-Component Characterization of Strain Rate Sensitivity in Pharmaceutical Materials. Doctoral dissertation, Duquense University, Pittsburgh, PA, 2015.

[39] Ryshkewitch E. (1953). Compression
strength of porous sintered alumina and zirconia: 9th communication
to ceramography. J. Am. Ceram. Soc..

[40] Duckworth W. (1953). Discussion
of Ryshkewitch paper by Winston Duckworth. J.
Am. Ceram. Soc..

[41] Eftaiha A. a. F., El-Barghouthi M. I., Rashid I. S., Al-Remawi M. M., Saleh A. I., Badwan A. A. (2009). Compressibility
and compactibility
studies of chitosan, xanthan gum, and their mixtures. J. Mater. Sci..

[42] Tye C. K., Sun C. C., Amidon G. E. (2005). Evaluation of the effects of tableting
speed on the relationships between compaction pressure, tablet tensile
strength, and tablet solid fraction. J. Pharm.
Sci..

[43] Mishra, S. M. Investigation of compaction behavior of pharmaceutical powders: an elucidation based on percolation theory. Doctoral dissertation, St. John’s University, Queens, NY, 2019.

[44] Rohatagi, A. *WebPlotDigitizer* ^ *®* ^. Copyright: 2010–2024. https://automeris.io/wpd4/(accessed March 28, 2023).

[45] Yu W., Liao L., Bharadwaj R., Hancock B. C. (2017). What is the “typical”
particle shape of active pharmaceutical ingredients?. Powder Technol..

[46] Merkus, H. G. Microscopy and Image Analysis. In Particle Size Measurements: Fundamentals, Practice, Quality; Springer Netherlands, 2009; pp 195–217.

