# Supplementary material for: Interaction-Based Model to Predict Tensile Strength of Compacted Mixtures from Individual Component Data
Source: Mol Pharm. 2025 Jul 14;22(8):5050–61. doi: 10.1021/acs.molpharmaceut.5c00709 (PMC12326345; doi:10.1021/acs.molpharmaceut.5c00709)
Supplement: Supplementary file 1 [file mp5c00709_si_001.pdf]

Supporting information 1

Interaction-based Model to Predict Tensile Strength of Compacted Mixtures from Individual Component Data

*Pradeep Valekar and Ira S. Buckner**

Graduate School of Pharmaceutical Sciences, Duquesne University, Pittsburgh, Pennsylvania 15282, United States.

***Corresponding Author**

Ira S. Buckner – Graduate School of Pharmaceutical Sciences, Duquesne University, Pittsburgh, PA 12582, Unites States, E-mail: buckneri@duq.edu

Identification of model binary mixtures to assess the prediction performance of interactions-based models.

Five common tableting excipients having similar particle size and aspect ratio were used in this study. Figure S1 shows the overlay of the compactibility profiles of these materials. The materials were selected to represent a range of different deformation behaviors. Avicel® PH200 and CombiLac® represented predominantly plastic materials. Lactose 316® and Emcompress® were selected as brittle materials. Lastly, Lycatab® PGS served as a viscoelastic material.

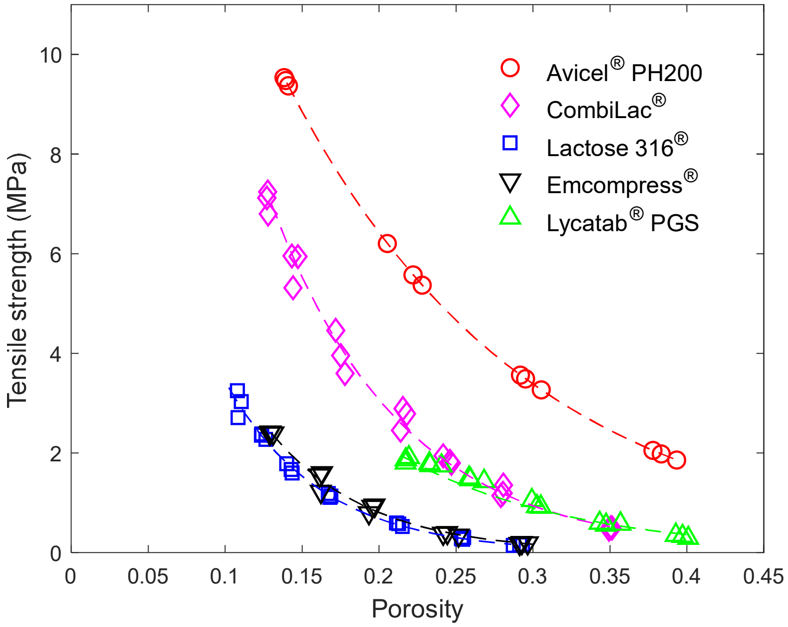

Figure S1. Compactibility profiles of pure components. The dashed lines represent the fit to the Ryshkewitch-Duckworth equation[1, 2] for the corresponding material.

Five pure components gave 10 binary mixture combinations, as shown in Table S1. The binary mixtures were prepared at the 75-25, 50-50, and 25-75 volume fraction ratios. All materials and mixtures were processed and characterized as per the methodology described in the manuscript. The tensile strength and volume fraction of the pure components were used in interactions-based

models to predict the tensile strength of binary mixtures. The predicted strength was compared to measure mixture strength to assess the performance of all models. To understand the observed results, the binary mixtures are numbered in ascending order of ratio of the tensile strength (σ_1/σ_2) of components at three porosities in Table S1. Mixture component 1 is a stronger material, while component 2 is a weaker material.

Table S1. Details of binary mixtures

Mixture #	Component 1	Component 2	σ_1/σ_2		
			ε = 0.15	ε = 0.20	ε = 0.25
1	Emcompress®	Lactose 316®	1.1	1.2	1.2
2	CombiLac®	Lycatab® PGS	1.6	1.4	1.2
3	Avicel® PH 200	CombiLac®	1.6	2.1	2.7
4	Lycatab® PGS	Emcompress®	2.0	2.8	3.8
5	Avicel® PH 200	Lycatab® PGS	2.5	2.9	3.3
6	Lycatab® PGS	Lactose 316®	2.3	3.3	4.7
7	CombiLac®	Emcompress®	3.1	3.8	4.6
8	CombiLac®	Lactose 316®	3.6	4.5	5.6
9	Avicel® PH 200	Emcompress®	5.0	8.0	12.6
10	Avicel® PH 200	Lactose 316®	5.8	9.4	15.4

Figures S2 and S3 show the overlay of tensile strengths predicted by different models and measured tensile strengths of all binary mixtures at 75-25 compositions. The pure component strengths are also overlaid in the plots for reference. For the mixtures containing both components

with similar tensile strengths, the predictions by all models converged. This behavior was illustrated by mixtures 1 and 2. For these mixtures, any model can be used to predict the strength.

As the strength difference between components grew gradually as displayed by mixtures 3 to 10, the predictions by different models started to diverge. Mixtures 9 and 10 showed the highest strength difference among all. In these mixtures, the predictions by different models showed the highest divergence. The same trends were also observed for the 25-75 and 50-50 compositions (data not shown here).

The main conclusions from this study are as follows.

1) For binary mixtures containing components with similar strengths, any model can be used to predict the mixture strength.
2) The binary mixtures containing components with higher strength differences are the model mixtures to assess the prediction performance of interactions-based models.

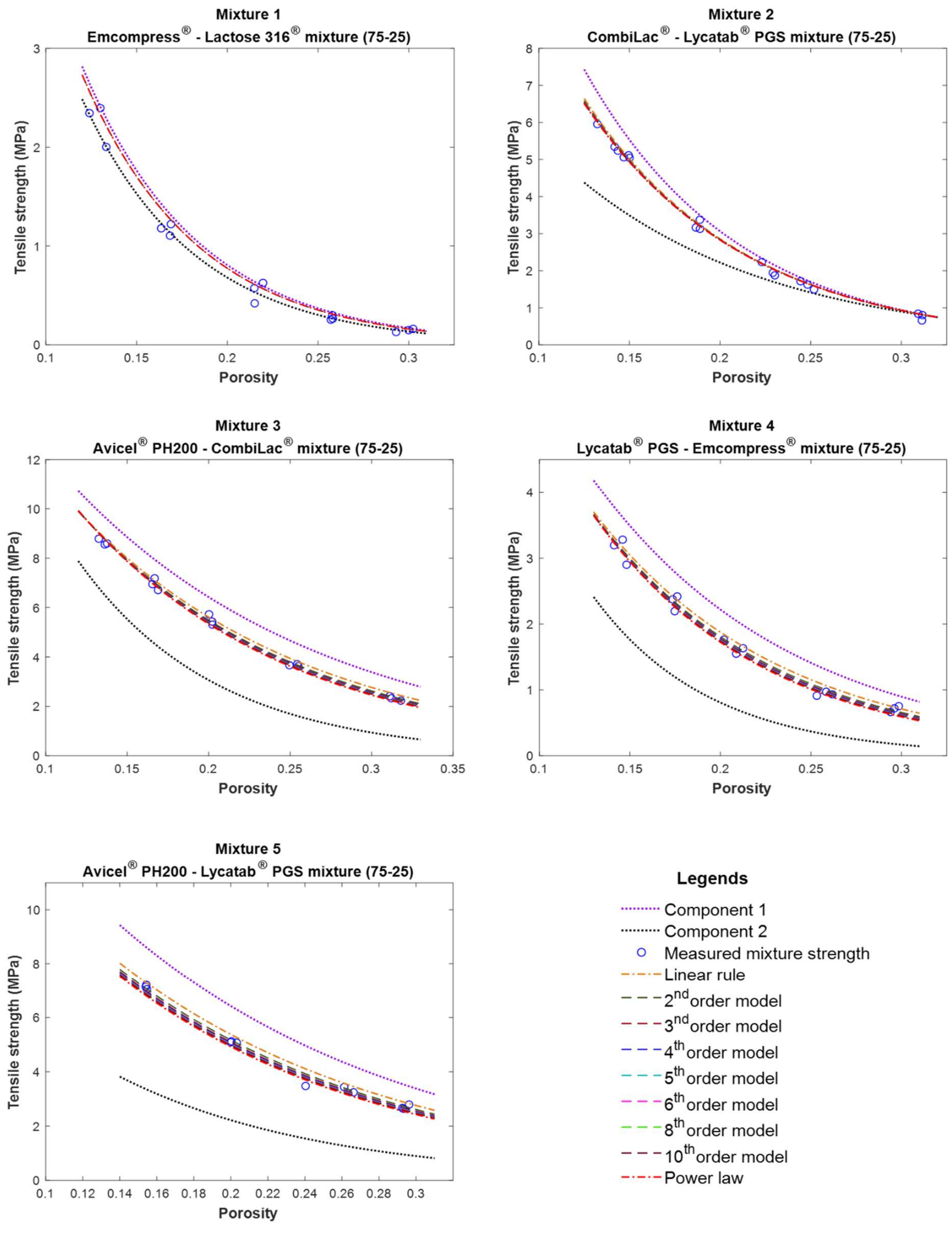

Figure S2. Overlay of measured and predicted strength by different models for mixtures 1 to 5

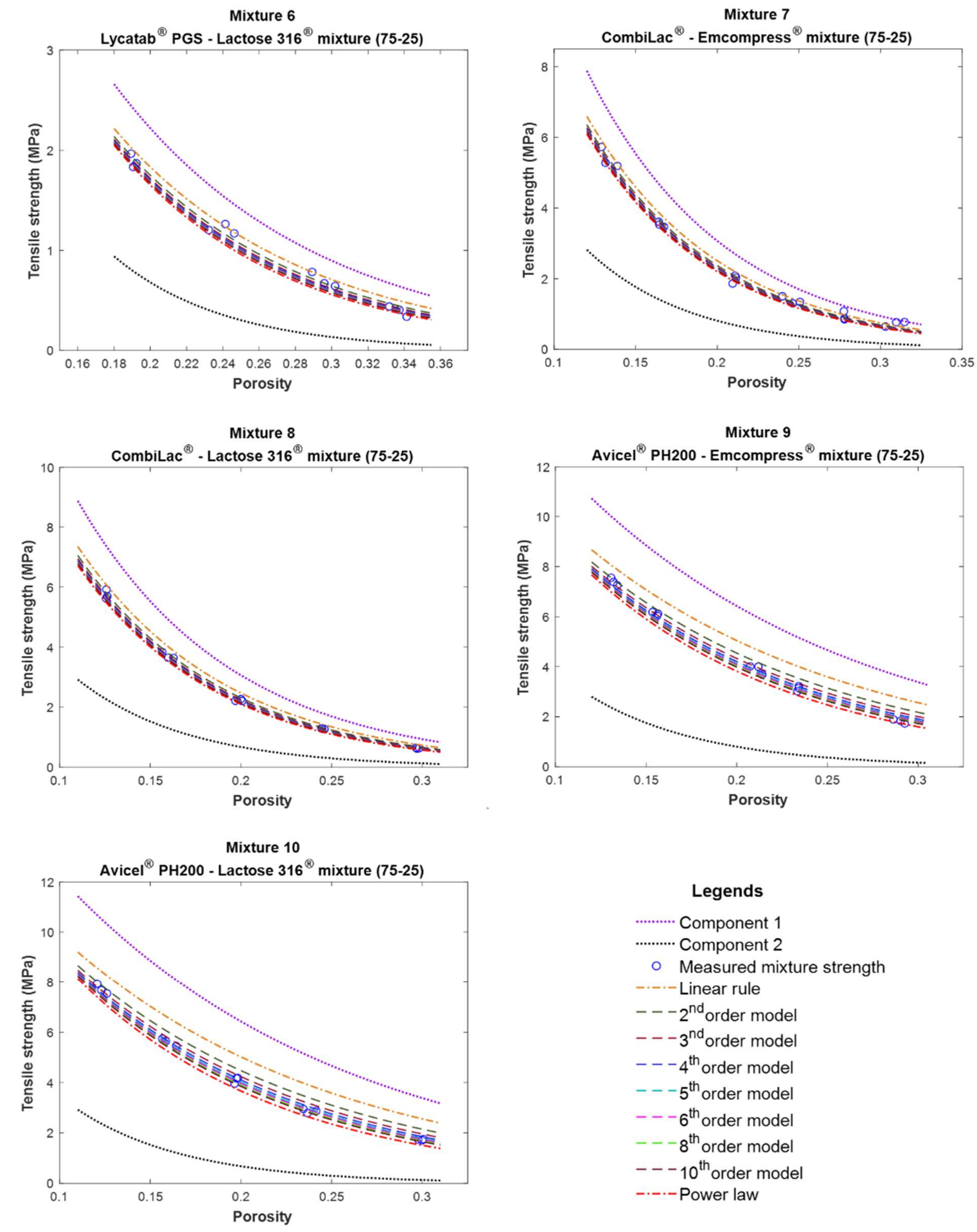

Figure S3. Overlay of measured and predicted strength by different models for mixtures 6 to 10

References

(1) Duckworth, W. Discussion of Ryshkewitch paper by Winston Duckworth. *J. Am. Ceram. Soc.* **1953**, *36*, 68-69.

(2) Ryshkewitch, E. Compression strength of porous sintered alumina and zirconia: 9th communication to ceramography. *J. Am. Ceram. Soc.* **1953**, *36* (2), 65-68.
